# Comparative analysis of creative problem solving tasks across age groups using modular cube robotics

**DOI:** 10.3389/frobt.2024.1497511

**Published:** 2024-12-13

**Authors:** Mehedi Hasan Anik, Margarida Romero

**Affiliations:** ^1^ MSc SmartEdTech, Université Côte d’Azur, Nice, France; ^2^ Science, Mathematics, and Technology Education (SMTE) Department, Institute of Education and Research, University of Dhaka, Dhaka, Bangladesh

**Keywords:** modular robotic cubes, creative problem solving (CPS), dual-process framework, divergent thinking, ill-defined creative problem, educational technology, stem education

## Abstract

Creative Problem Solving (CPS) is an important competency when using digital artifacts for educational purposes. Using a dual-process approach, this study examines the divergent thinking scores (fluidity, flexibility, and originality) and problem-solving speed in CPS of different age groups. Participants engaged in CreaCube CPS tasks with educational robotics for two consecutive instances, with performance analyzed to explore the influence of prior experience and creative intentions. In the first instance, infants and children demonstrated greater originality compared to seniors, solving problems quickly but with less originality. In the second instance, teens, young adults, and seniors showed enhanced originality. The results highlight trends influenced by prior experience and creative intentions, emphasizing the need for customized instructions with modular robotics to improve CPS across the lifespan.

## 1 Introduction

The integration of educational robotics into K-12 and adult learning environments has the potential to foster problem-solving skills in diverse age groups, including individuals with disabilities (Kirksey et al., 2023). Educational robotics offers hands-on, interactive opportunities for engaging in Creative Problem Solving (CPS) activities, facilitating the exploration of complex concepts through active problem-solving. Since the late 1980s, robotics has been employed as both a subject and a pedagogical tool across various academic disciplines, including computer science and engineering, making it a versatile resource for promoting CPS skills in learners (Karatrantou and Panagiotakopoulos, 2011).

The introduction of robotics in educational settings enhances students’ problem-solving efficacy by encouraging deductive reasoning and the practical application of theoretical knowledge (Atmatzidou and Demetriadis, 2017). Robotics-based tasks, such as those using modular Cubelets or Lego robotics, enable students to develop scientific skills, positive attitudes toward technology, and an appreciation for the creativity involved in constructing functional systems (Jaipal-Jamani and Angeli, 2017; Gomoll et al., 2016). However, despite its potential, students still encounter few opportunities to apply CPS skills to open-ended challenges within formal educational settings (Jonassen, 2009; Zhang and Zhu, 2022).

The CreaCube task, which involves constructing functional robotic systems using modular Cubelets, has emerged as an innovative tool for studying CPS across different age groups. It allows researchers to assess components of divergent thinking, including fluidity, flexibility, and originality (Kalmpourtzis and Romero, 2022). While prior research has predominantly examined CPS within single age groups, a comprehensive understanding of CPS abilities across developmental stages remains underexplored. To deepen this understanding, this study incorporates the dual-process framework, which distinguishes between fast, intuitive thinking (System 1) and slow, analytical thinking (System 2) (Kahneman, 2011). Within CPS, System 1 is responsible for rapid idea generation, while System 2 refines and evaluates those ideas. Leroy and Romero (2022) applied this framework to educational robotics, finding that creative outcomes often emerge from an interplay between these cognitive processes, where initial divergent ideas from System 1 are refined by System 2. This study builds on their approach by examining how participants of different ages balance these cognitive processes in CreaCube tasks, aiming to identify how CPS strategies evolve across the lifespan.

Addressing this gap, the current study explores CPS differences among various age groups, from infants to seniors, through performance analysis in the CreaCube task. By integrating the dual-process framework, this research not only examines age-related variations in divergent and convergent thinking but also sheds light on the influence of prior experience and creative intentions on CPS performance in educational robotics. Findings from this study will contribute a developmental perspective to educational robotics research, supporting the design of age-appropriate interventions that foster creativity and problem-solving skills through robotics-based learning environments.

## 2 Problem-solving in educational robotics

This study aims to analyze the CPS process in educational robotics within the CreaCube robotics task. The nature of CPS across different age groups was explored by comparing fluidity, flexibility, and originality across two instances of the CreaCube task. Since participants were unfamiliar with the CreaCube task, their initial engagement was guided by creative intentions, resulting in longer time spent on the first task compared to the second. Consequently, all age cohorts exhibited greater fluidity and flexibility during the first instance, aligning with previous findings that emphasize the role of creative intention in fostering longer engagement and higher instances of divergent thinking (Verner et al., 2022; Pyatt and Sims, 2012). Verner et al. (2022) highlight the potential of educational robotics, such as modular cubes, to enhance students’ engagement and problem-solving by offering hands-on learning experiences that support active exploration and creativity in STEM education.

As observed in this study, infants and children displayed unintended creative outcomes with higher fluidity and flexibility in the first task, whereas older cohorts showed conservative outcomes with lower originality. This pattern aligns with research indicating that younger students often exhibit more intuitive and flexible problem-solving strategies compared to the more analytical approach of older students (Syawaludin et al., 2019; Lin and Chiu, 2004). Furthermore, the distinct CPS strategies adopted by different age groups align with Lin and Chiu’s (2004) findings, which suggest that elementary students lean toward intuitive and trial-based problem-solving, while older students adopt structured and goal-oriented strategies. This suggests that the CreaCube task may effectively engage younger children’s inherent flexibility, as noted in studies on modular robotics that show hands-on learning platforms encourage a range of strategies in CPS (Kalmpourtzis and Romero, 2022).

In the second instance, participants approached the task with prior experience but without creative instruction, which led to a dichotomy of intentions. Some participants repeated previous solutions (conservative behavior), while others attempted new solutions (creative intention). Such patterns are consistent with findings that technology-supported problem-solving can foster varying approaches, with more experienced participants often displaying conservative behaviors due to familiarity with the task (Roberts et al., 2018; Christensen et al., 2019). Roberts et al. (2018) emphasize that robotic activities can support differentiated problem-solving approaches depending on the learner’s prior experience and age, with elementary students benefiting from exploration-based learning and older students showing preference for structured problem-solving. In our study, teens, young adults, and seniors exhibited increased originality in the second task, suggesting a self-motivated shift toward creative intention, which may point to the importance of experience-driven adaptability in CPS (Choi et al., 2013).

Overall, while preliminary and exploratory, these findings advance understanding of age-related CPS patterns in educational robotics through a dual-process framework, considering how creative behavior emerges in the absence of explicit instruction but with prior experience. The literature supports that technology-enhanced problem-solving, when aligned with cognitive developmental stages, can foster varied approaches in CPS, potentially informing future educational strategies for diverse age groups.

## 3 Divergent and convergent thinking in creative problem solving

**FIGURE 1:**
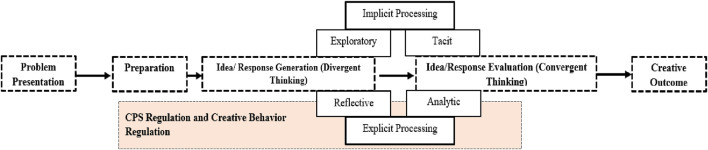
CPS Process regulation of Divergent and Convergent Thinking.

In CPS, participants engage in two crucial processes, Divergent Thinking (DT) and Convergent Thinking (CT) (Figure 1), demonstrating a specific creative intention and endurance in preserving it throughout the instances of the CreaCube task (Leroy and Romero, 2021). The process of idea generation relies on divergent thinking, a fast, implicit system grounded in prior information and actions. In contrast, idea evaluation through convergent thinking demands more effort due to the learner’s persistence and motivational orientation toward desired outcomes. Ultimately, the creative outcome, if predefined, guides implicit processing and encourages further creative behaviors through explicit processing, ultimately leading to the desired outcome (Romero, 2022).

## 4 Creative problem solving through educational robotics tasks

Research on Creative Problem Solving (CPS) across educational levels reveals distinct approaches among students. Elementary students tend to approach problems intuitively, relying less on abstract reasoning, which may pose challenges (Syawaludin et al., 2019). In contrast, middle schoolers, as noted by Lin and Chiu (2004), demonstrate a tendency to employ more systematic problem-solving strategies. High school students, according to Choi et al. (2013), showcase higher-order thinking skills and favor a more analytical approach to tackling problems.

Several studies investigating the incorporation of robotics activities into academic programs provide insight into the learning patterns and developmental stages observed across students of all different ages and genders. Sullivan and Bers (2016) discovered that kindergarten kids could understand the basics of robotics and programming after putting an 8-week robotics curriculum into practice. Particularly, the older children learned increasingly more complex concepts in the same amount of time by using the same robotics kit. Jackson et al. (2019) observed gender-specific traits during LEGO robotics contests and discovered that both boys and girls enjoy participating in robotics activities. Although the boys place more emphasis on acquiring technical abilities in their reporting, while the ladies place more emphasis on reporting on non-technical aspects (such as cooperation and communication skills). Chevalier et al. (2020) compared two groups of elementary school children participating in a Thymio-educational robotics exercise and discovered that kids often exhibit *“trial-and-error behavior”* in the absence of adequate systematic instruction before the task.

Investigations into the incorporation of robotics in science classrooms unveil diverse experiences among students. Roberts et al. (2018) observed that elementary students exhibit enthusiasm and curiosity when engaging with robotics, benefiting from hands-on learning opportunities. Middle school students, as reported in the same study, appreciate the teamwork and problem-solving challenges associated with programming robots. Conversely, Christensen et al. (2019) found that high school students value robotics for its practical applications and potential alignment with future careers.

Observing students engaging in CreaCube robotics activities with their dyad, Cassone et al. (2021) found that participants employing more time on problem-solving activities tend to show more innovative configurations. Within the CreaCube repeated activities, Leroy and Romero (2022) explored that most participants tend to show less creativity in the second instance of the task due to the development of a conservative mindset after the first instance of the CreaCube task. Hereby, participants complete the second instance of the task very quickly, and in most cases, produce conservative outcomes. Therefore, participants’ creative intention and proper creativity instructions are essential to get creative outcomes from the participants in both repeated instances of the CreaCube task. Moreover, Leroy et al. (2023) compared the Divergent Thinking (DT) components (e.g., fluidity, flexibility, and originality) in the Alternate Uses Test (AUT) of familiar objects (e.g., chair, can, and box) and CreaCube task using unfamiliar cube objects and found that unfamiliarity leads the participants towards trial-error behavior and as a result less fluency, flexibility, and originality for the later instance (though the differences are not significant). Romero’s (2022) study involving a range of age groups shows that children and Seniors exhibit greater fluidity in creative elements but require more time investment. It's interesting to see that children score higher than other age groups in terms of fluidity, flexibility, and originality, but they also devote more time to their CPS.

## 5 Purpose of the study

The study aims to examine and compare individuals’ ill-defined CpS capacities across different age brackets when engaging with modular cube robotics. This research evaluates the solutions’ time, fluidity, flexibility, and originali*ty* demonstrated by infants, children, teens, young adults, and seniors. By scrutinizing these diverse age groups, the study seeks to uncover patterns, variations, or distinctions in problem-solving techniques concerning modular cube robotics from the lens of the dual-process framework. The study aims to study age-related CPS employed with modular robotics. Specifically, the study aims to identify and analyze age-related patterns in problem-solving strategies employed with modular cube robotics to uncover variations among age cohorts and between the two instances of the CreaCube task.

## 6 Research questions

The research question aims to identify the quantitative differences in CPS duration, fluidity, flexibility, and originality exhibited by participants of different age groups in the first instance and the second instance of the CreaCube task. Based on the purpose and scope of the study described, two research questions that align with the goals of assessing age-related differences in Creative Problem Solving (CPS) with modular cube robotics and are followed in this study are:

RQ.01: How do age-related differences impact the Divergent Thinking components—time, fluidity, flexibility, and originality—within the dual-process framework of Creative Problem Solving (CPS) when participants engage with the CreaCube task across different age groups?
RQ.02: What changes in CPS outcomes, particularly in terms of time, fluidity, flexibility, and originality, can be observed between the first and second instances of the CreaCube task, and how do these changes align with creative and conservative intentions across different age groups?

## 7 Theoretical framework

### 7.1 Dual process model for conservative and creative intentions and behavior

**FIGURE 2:**
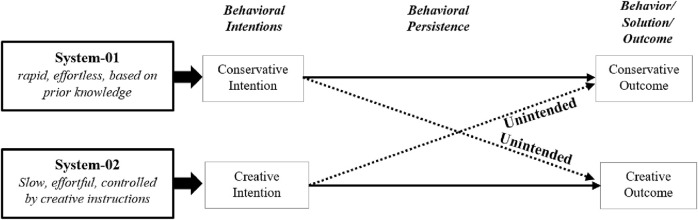
Dual process framework for creative problem solving.

Houdé and Borst (2014), and Kahneman (2011) identified two parallel opposed cognitive systems to explain the dual process model. As described in the dual process models (Figure 2), the First System (S1) embodies a cognitive process characterized by rapid, automatic, and intuitive thinking. It is primarily driven by prior knowledge and familiar associations, leading to quick, habitual responses to tasks or problems. This system enables individuals to rely on well-established solutions and pre-existing mental frameworks to streamline decision-making and problem-solving processes. On the other hand, the Second System (S2) represents a contrasting cognitive process characterized by deliberate, controlled, and effortful thinking. It operates as a counterbalance to the First System by engaging in more reflective, conscious, and analytical processing. The second system (S2) involves inhibiting automatic responses driven by familiar associations and actively exploring novel or unconventional pathways to problem-solving.

Based on the model, Leroy and Romero (2022) proposed a dual-process framework (Figure 1) for describing the creative process in the educational robotics context where both systems have been considered to produce creative outcomes. The framework proposed how conservative and creative intentions influence participants’ behavior towards creating creative and conservative outcomes in solving ill-defined creative problems. They observed that any creative solution is a result of an effortful regulatory procedure driven by creative intention. To accelerate creative intention among the participants, it is imminent to learn conservative behavior of solving the task with an already known solution. Such conservative intention can persist throughout the next events if the participants feel the task is difficult. Although, giving creative instructions for the second task has the potential to promote creative intention, which can, in turn, encourage participants to display creative behaviors and outcomes (Paulus et al., 2011).

### 7.2 Divergent thinking

According to Guilford (1967) and Kharkhurin (2009), *divergent thinking* (DT) is comprised of four fundamental qualities that are essential to the creative thinking process: *fluidity* (fluency), flexibility, originality, and elaboration. Together, these attributes influence how people generate, investigate, and implement ideas when they are faced with creative or problem-solving activities.

*Fluidity*- In the context of divergent thinking, fluidity—embodies the quickness with which a diverse range of concepts or solutions can be generated. Fluid people can generate a lot of ideas swiftly, which allows for an unrestricted flow of creativity. When applied to CPS situations, fluidity encourages deep brainstorming and exposes people to a wide range of possibilities. The core question to identify fluidity is- “How many ideas?”

*Flexibility*- Being flexible means having the capacity to accept several methods or points of view simultaneously when solving a particular problem. Flexibility experts demonstrate versatility and an openness to accepting different viewpoints. This characteristic encourages the exploration of unconventional solutions that support creative approaches to problem-solving. The representative question from flexibility is- “How many different ideas.?”

*Originality*- The tendency to come up with unique ideas or solutions that deviate from conventional concepts or solutions is what is meant to be embodied in originality. People who are creative present new ideas that are very different from the mainstream common ideologies. Accepting uniqueness enables people to go beyond preconceived notions in search of innovative solutions. To measure originality, the generic question to be posed is- “how many unique solutions.?”

*Elaboration-* Elaboration is the process of comprehensive expansion of ideas and carrying them out in a way that makes sense. In the process of real-world problem solving (Okuda et al., 1991), elaboration is essential because it helps the person who is skilled at it to convert imaginative concepts into workable solutions that precisely and successfully solve problems in the real world. In some educational CPS, elaboration is not considered because the process and results do not allow to develop an expansion of ideas.

To measure the Divergent Thinking components quantitatively in this study, *fluidity* is assessed by counting the total number of distinct configurations each participant generates during the CreaCube task; for example, if a participant attempts five different arrangements of cubes, their fluidity score is 5, reflecting idea generation breadth. *Flexibility* is measured by tracking the quantity of distinct strategies participants employ, such as shifting from stacking cubes vertically to arranging them side-by-side; each distinct strategy shift adds to the flexibility count (e.g., three strategy changes yield a flexibility score of 3), indicating adaptability. Originality is measured by counting the number of unique solutions that differ from others’ attempts; if a participant creates two configurations that are distinct from any others observed, they receive an originality score of 2, reflecting the novelty of their approach. These quantitative measures—number of configurations for fluidity, strategy shifts for flexibility, and unique solutions for originality—provide a structured, numerical assessment of creative problem-solving attributes across participants.

## 8 Conceptual framework

Leroy and Romero (2022) insisted that towards creating creative solutions, participants solving educational robotics problems should grow creative intentions. Before that, inhibiting a certain degree of prior experience with the task is necessary for being effective in solving the problem creatively where instruction to be creative can go side by side to accelerate creative intentions (Chua and Iyengar, 2008). According to Leroy and Romero (2022), Conservative intention leads participants toward no or less creative solutions and encourages the participant to provide less effort (less time, fewer try-outs) and as a result, the participant solves the problem based on the prior experience or previous solution, thus, creates “conservative” or “non-intentional creative” outcome. In contrast, creative intention promotes more effort (more time, more try-outs) and finally leads towards “creative” or “non-intentional conservative” solutions.

**FIGURE 3:**
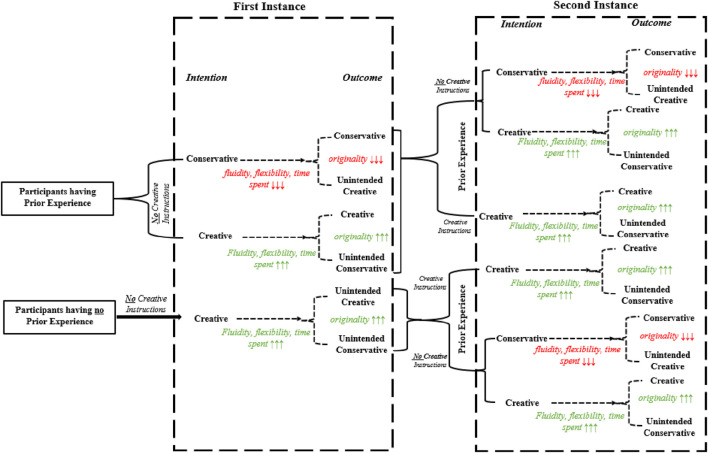
Conceptual framework of the study: Dual-process creativity model for educational robotics activities.

Furthermore, Romero (2022) explained that the more fluid and flexible participants produce more creative outcomes or originality of the outcomes. Therefore, the less fluid and flexible behavior of the participants results in less originality. Theoretically, participants will need to invest more time to show more fluency and flexibility by thinking and trying out different ideas. Moreover, we can find a link between fluidity and flexibility, time spent with conservative/creative intentions, and originality with conservative/creative outcomes. Our study aims at providing participants to engage in repeated instances. To think about the influence of prior knowledge, can influence the participants having prior knowledge in a similar task and participants having no prior experience regarding the task for the first instance. Additionally, the fact of gaining prior experience during the first instance is also considered to be influential while engaging in the second instance of a similar task. Hereby, the conceptual framework of the study is presented in Figure 3

## 9 Methodology

### 9.1 Research design

This study adopts a cross-sectional design to compare the problem-solving capacities of individuals from various age groups engaging with modular cube robotics. A quantitative cross-sectional approach is employed to collect and analyze data regarding problem-solving speed (time spent), fluidity, flexibility, and originality exhibited by participants of different ages at a particular time (Johnson and Christensen, 2019).

### 9.2 Study context

Using the CreaCube task, we assess the Divergent Thinking components of fluidity, flexibility, and originality to understand the regulation process within the CPS (Romero, 2022) from the lens of a dual-process system. The elaboration criteria of divergent thinking are not considered due to limitations in the expansion of possible configurations within the CreaCube task.

### 9.3 Participants

The study recruited participants across diverse age brackets, specifically infants, children, teens, young adults, and seniors, to capture a range of developmental stages relevant to Creative Problem Solving (CPS) abilities. A purposive convenience sampling method was employed, selecting individuals based on availability and interest in the task (Creswell, 2020). Participants were engaged during the Science Festival 2023 at the Antibes Science and Innovation Village on October 21, allowing attendees of various ages to experience the modular robotics task.

The chosen age brackets—Infants (5–6 years old), Children (7–12 years), Teens (13–18 years), Young Adults (19–29 years), and Seniors (60–79 years)—were selected to represent broad developmental phases that might influence CPS strategies and divergent thinking attributes across different age groups (Romero, 2022). This classification enables a focus on cognitive and creative differences across early childhood, adolescence, young adulthood, and later adulthood. Although this study used these specific groupings, alternative classifications, such as grouping by educational levels (e.g., elementary, middle school, high school), could provide additional insights into age-related CPS variations within specific learning contexts. Notably, none of the participants had prior experience with modular cubelet robotics. All participants provided consent for recording, and their demographic details are as follows (Table 1).

**TABLE 1:** Demographic information of the participants.

Age groups	No. of participants	No. of male participants	No. of female participants
Infants (0 to 6 years old)	2	-	2
children (7 to 12 years old)	9	3	6
teens (13 to 18 years old)	2	-	2
young adults (19 to 29 years old)	2	2	-
Seniors (60 to 79 years old)	2	1	1
Total	**17**	**6**	**11**

Bold values represent the total number of participants and their distribution by gender across all age groups.

### 9.4 Procedure

**FIGURE 4:**
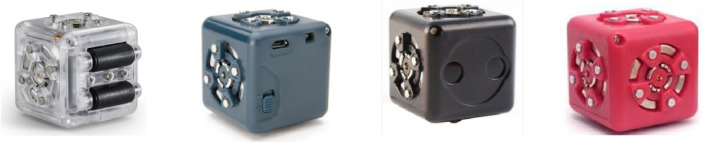
Four modular cubelets offered to solve ill-defined creative problem.

The CreaCube task is a CPS task designed for participants to construct an independently moving vehicle using four modular robotic cubes selected from the Cubelets toolkit (Romero and Barma, 2022). Each modular robotic cube serves a unique function, identified by color: the *white cube* with wheels provides “drive affordance,” the *blue cube* functions as an energy source powered by a switch button, the *black cube* includes a “distance sensor affordance,” and the *red cube* embodies an “inverter signal affordance” (Kalmpourtzis and Romero, 2022) (Figure 4). Through this ill-defined CPS task, mediated by tangible interactive technologies, participants engage in creative problem-solving by generating, evaluating, inhibiting, and transforming ideas across the CPS process (Romero, 2022).

**FIGURE 5:**
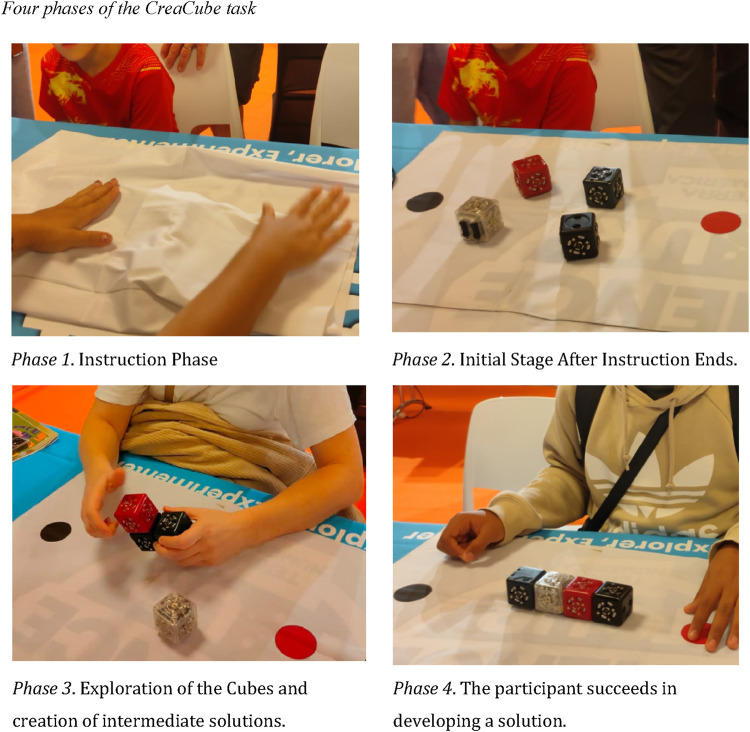
Four phases of the CreaCube task. Phase 1. Instruction Phase. *Ph*ase 2. Initial Stage After Instruction Ends. Phase 3. Exploration of the Cubes and creation of intermediate solutions. Phase 4. The participant succeeds in developing a solution.

This study aimed to guide the cubes from a starting red point to a final black point without time constraints, allowing participants the freedom to explore. Task 1 and Task 2 were identical in nature and complexity for all age groups, designed to provide a consistent basis for observing CPS abilities without introducing variations between trials or age-related adjustments. Participants followed a set protocol: initially, the cubes were concealed and gradually revealed as the participant listened to instructions. During this time, they explored the cubes’ features and experimented with various configurations to construct an autonomous moving vehicle (Romero et al., 2019). Participants arranged the four cubes in diverse ways to achieve the task’s objective without using any language or drawings to communicate their ideas, engaging directly with the tangible robotics components to solve the challenge (Figure 5).

### 9.5 Data collection

The data collection process involved a comprehensive analysis of video recordings and observational notes to evaluate participants’ performance during the CreaCube task, with a focus on four key metrics: time, fluidity, flexibility, and originality. Video recording was central to capturing each participant’s engagement with the task, and a total of 17 videos were taken to ensure comprehensive data coverage. Cameras were strategically positioned face-to-face to capture participants’ interactions with the modular cubes, allowing for accurate observation of hand movements, configuration adjustments, and overall problem-solving behavior.

The timing of each participant’s task performance was measured precisely, beginning from the moment they received initial instructions and the cubes were revealed (start point) to when they successfully configured a moving structure with the cubes (endpoint). For *fluidity*, if a participant tries five different cube configurations, their fluidity score is 5, indicating the number of ideas generated. For *flexibility*, if a participant shifts strategies three times—such as switching from stacking cubes vertically to arranging them side-by-side and then diagonally—their flexibility score is 3, showing adaptability in approach. For *originality*, if a participant creates two configurations distinct from any other attempts, their originality score is 2, representing the novelty of their solutions.

To maintain consistency in coding, a detailed coding system was developed, and two independent coders analyzed the recordings, achieving high inter-rater reliability through repeated checks. Observational notes further supplemented the video analysis, capturing non-verbal cues, such as facial expressions and body language, which provided qualitative insights into the participants’ creative intentions. Although direct interviews were not conducted, these qualitative observations enriched the data, offering additional context for understanding participants’ problem-solving processes and intentions.

### 9.6 Ethical considerations

The study has been approved by the ethical committee of Université Côte d’Azur (2019-6). All participants provided informed consent to record videos, and the study complied with ethical standards. Anonymity and confidentiality were guaranteed when managing and reporting data (Creswell, 2020).

## 10 Results

The results of the study are organized and aimed to inform the research questions of this study, which aims to identify age differences in divergent thinking and duration in each of the two instances of the CPS task. For the analysis of age differences in CPS duration, fluidity, flexibility, and originality through the different age groups.

### 10.1 Analysis of the first instance of the CreaCube task

Analysis of the participant’s performance in the first instance of the task showed that different age groups’ interactions with modular cube robotics followed diverse patterns. Compared to teens and younger adults, infants, children, and Seniors showed an affinity to devote more time to problem-solving activities (Tables 2, 3).

**TABLE 2:** Analysis of the first instance of the CreaCube task.

Age groups = N		Time (seconds)	Fluidity	Flexibility	Originality
Infants= 2	Mean	150.5	3.5	2	1
	SD	67.18	0.71	1.41	0
Children = 9	Mean	93.33	2.67	1.11	0.11
	SD	70.90	1.87	0.33	0.33
Teen = 2	Mean	111	2	1	0
	SD	66.47	1.41	0	0
Young Adult = 2	Mean	54	1	1	0
	SD	2.83	0	0	0
Senior = 2	Mean	96	1.5	1.5	0
	SD	97.58	0.71	0.71	0
Total = 17	Mean	**97.82**	**2.35**	**1.24**	**0.18**
	SD	**65.48**	**1.58**	**0.56**	**0.39**

The second last row labeled “Mean” represents the average values for time, fluidity, flexibility, and originality across all participants and age groups. The last row labeled “SD” represents the average standard deviation for time, fluidity, flexibility, and originality across all participants and age groups.

**TABLE 3:** Individual data for infants, teen, young adults, and seniors at Activity-01.

Participants	Gender	Time (seconds)	Fluidity	Flexibility	Originality
Infant-01	Female	198	4	1	1
Infant-01	Female	103	3	3	1
Teen-01	Female	64	1	1	0
Teen-02	Female	158	3	1	0
Young Adult-01	Male	52	1	1	0
Young Adult-02	Male	56	1	1	0
Senior-01	Female	165	1	1	0
Senior-02	Male	27	2	2	0
Total = 8	Male = 3, Female = 5				

It is noteworthy that infants and children demonstrated a greater degree of fluidity in their methods for addressing problems, indicating a tendency to come up with diverse ideas. In addition, Seniors and infants showed far more flexibility than other age groups, highlighting their ability to think of many ways to solve the ill-defined problems that were put forth. One noteworthy finding was that infants’ and children’s solutions could prove originality, which was not the case with the other age groups’ problem-solving results from the first instance of the task.

### 10.2 Analysis of the second instance of the CreaCube task

When it came to the second instance of the task, infants and seniors tended to spend less time with it, indicating a preference for quick problem-solving through recall, whereas teens and young adults spent more time experimenting and exploring different possibilities. Consequently, compared to other age groups, infants and Seniors showed less flexibility in their approaches to problem-solving, indicating a smaller capacity for coming up with original ideas. As a result, compared to other age groups, infants and children had much less flexibility (m_infants_ = 0.5; m_children_ = 0.89), suggesting a lower inclination to explore other strategies. Interestingly, the solutions of teens, young adults, and seniors showed a greater degree of originality (m_teens_ = 0.5; m_youngAdults_ = 1; m_seniors_ = 0.5) than those of children (m_children_ = 0.11), whose originality stayed consistent with the first instance of the task (Tables 4, 5).

**TABLE 4:** Analysis of the second instance of the CreaCube task.

Age Groups = N		Time (seconds)	Fluidity	Flexibility	Originality
Infants= 2	Mean	21	0.5	0.5	0
	SD	29.70	0.71	0.71	0
Children = 9	Mean	41	1.56	0.89	0.11
	SD	35.51	1.42	0.33	0.33
Teens = 2	Mean	42	1.5	1	0.5
	SD	15.56	0.71	0	0.71
Young Adults = 2	Mean	99	3	1.5	1
	SD	97.58	1.41	0.71	1.41
Seniors = 2	Mean	29.5	1	1	0.5
	SD	2.12	0	0	0.71
Total = 17	Mean	**48.06**	**1.71**	**1**	**0.29**
	SD	**44.75**	**1.40**	**0.5**	**0.59**

The second last row labeled “Mean” represents the average values for time, fluidity, flexibility, and originality across all participants and age groups. The last row labeled “SD” represents the average standard deviation for time, fluidity, flexibility, and originality across all participants and age groups.

**TABLE 5:** Individual data for infants, teen, young adults, and seniors at Activity-02.

Participants	Gender	Time (seconds)	Fluidity	Flexibility	Originality
Infant-01	Female	0	0	0	0
Infant-01	Female	107	4	2	0
Teen-01	Female	53	2	1	1
Teen-02	Female	31	1	1	0
Young Adult-01	Male	168	4	1	0
Young Adult-02	Male	168	4	1	0
Senior-01	Female	30	2	2	2
Senior-02	Male	28	1	1	1
Total = 8	Male = 3, Female = 5				

### 10.3 Comparative analysis of the two instances of the task

Comparing both instances of the task, we observe notable variations in how different age groups approached problem-solving with modular cube robots. Interestingly, there was a general tendency across all age groups to spend less time on the second instance of the task (m = 48.6 s) than on the first instance of the task (m = 97.82 s). Concerning divergent thinking, a notable finding for infants and seniors was that, when comparing the two instances, their methods of problem-solving were less fluid, flexible, and original in the second instance. The remaining age groups, on the other hand, displayed an opposite pattern, exhibiting higher degrees of originality, fluidity, and flexibility in the second instance of the task than they did in the first instance of the task. This difference illustrates a distinct behavior in the CPS process of infants, seniors, and other age cohorts between the two successive modular cube robotics instances.

## 11 Discussion

This preliminary study aims to explore the CPS process in educational robotics within the CreaCube robotics task. The nature of CPS across different age groups was examined by comparing fluidity, flexibility, and originality across two instances of the CreaCube task. Since participants were unfamiliar with the CreaCube task, their initial engagement appeared to be guided by creative intentions, resulting in a longer duration on the first task compared to the second. Consequently, all age cohorts tended to exhibit greater fluidity and flexibility during the first instance, supporting findings that suggest creative intention may encourage extended engagement and increased divergent thinking (Verner et al., 2022; Pyatt and Sims, 2012). Verner et al. (2022) highlight the potential of educational robotics, such as modular cubes, to enhance students’ engagement and problem-solving by providing hands-on learning experiences that support exploration and creativity in STEM education.

In this study, infants and children showed higher fluidity and flexibility in the first task, leading to unintended creative outcomes, whereas older cohorts appeared to show more conservative outcomes with lower originality. This observation aligns with research suggesting that younger students often display more intuitive and flexible problem-solving strategies compared to the more analytical approach observed in older students (Syawaludin et al., 2019; Lin and Chiu, 2004). Furthermore, the distinct CPS strategies seen across age groups resonate with Lin and Chiu’s (2004) findings that elementary students often employ trial-based problem-solving, while older students adopt more structured, goal-oriented strategies. While preliminary, this suggests that the CreaCube task may engage younger children’s inherent flexibility, consistent with studies on modular robotics that indicate hands-on platforms may encourage a diversity of problem-solving strategies (Kalmpourtzis and Romero, 2022).

**FIGURE 6:**
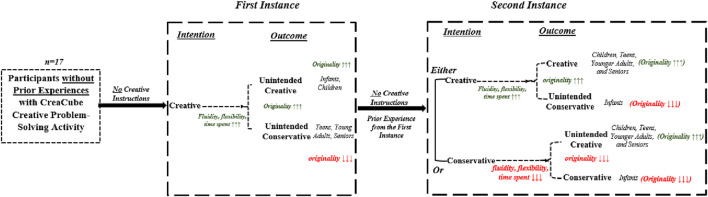
Visual representation of the findings through the lens of the dual-process model.

In the second instance, participants approached the task with prior experience but without additional creative instruction, which led to a mix of conservative and creative intentions. Some participants repeated previous solutions (conservative behavior), while others attempted new solutions (creative intention). This aligns with findings indicating that technology-supported problem-solving can promote varied approaches, with participants familiar with a task often adopting conservative behaviors (Roberts et al., 2018; Christensen et al., 2019). Roberts et al. (2018) suggest that robotic activities may foster differentiated problem-solving approaches based on the learner’s experience and age, with elementary students benefiting from exploration and older students gravitating toward structured problem-solving. In this study, teens, young adults, and seniors exhibited increased originality in the second task, potentially reflecting an experience-driven shift toward creative intention, a point that warrants further exploration (Choi et al., 2013). The findings have been visually presented through the lens of the dual-process model as follows (Figure 6).

Overall, while preliminary and exploratory, these findings provide initial insights into age-related CPS patterns in educational robotics through a dual-process framework, considering how creative behavior may emerge in the absence of explicit instruction but with prior experience. Literature supports that technology-enhanced problem-solving, when aligned with cognitive developmental stages, can foster varied approaches in CPS, which could inform future educational strategies for diverse age groups. However, given the limited scope of this study, these findings should be interpreted as trends rather than conclusive evidence, encouraging further research with a larger sample to confirm these observations.

## 12 Limitations

Given the study’s small sample size, statistical tests such as ANOVA or Kruskal–Wallis were not feasible due to insufficient statistical power. As a preliminary study, these findings should be interpreted as indicative trends rather than definitive group differences, providing a foundation for future research with a larger sample. Additionally, selecting participants from a science and innovation fair may introduce a sampling bias, as attendees are likely to have a pre-existing interest in STEM subjects, which may influence their engagement with the CreaCube task and affect the generalizability of results. The limited sample size further constrains broader generalizations, as it may not fully capture the diversity of approaches that participants might employ in the CreaCube task. Consequently, the study’s insights should be viewed as exploratory, emphasizing potential patterns in age-related CPS rather than conclusive evidence.

## 13 Implications of the study

The insights from this research study can be applied in other CPS learning activities, especially in those involving modular robotics. Teachers may use these findings to develop age-appropriate teaching strategies, not only in modular robotics but also in any context of CPS with manipulable artifacts. By aligning lesson plans with cognitive developmental stages and fostering enhanced creative problem-solving, educators can create targeted approaches tailored to the needs of different age groups. Additionally, those designing new technologies for educational settings can utilize these outcomes to create more engaging and effective science learning environments. The discussion based on the dual-process framework may further provide a theoretical basis that can serve as a hypothesis for future research, aiming to conclusively identify the distinct behaviors exhibited by different age groups during CPS tasks.

## 14 Conclusion

In educational robotics, modular robotics tools such as the CreaCube task offer a structured approach to studying CPS across various age groups. This study identified distinctive patterns in divergent thinking—fluidity, flexibility, and originality—between the first and second instances of the task. Participants generally spent less time on the second instance; however, originality scores for seniors increased from 0 to 0.5, indicating that prior experience may have varying effects on CPS outcomes across age groups. In contrast, other age groups showed heightened CPS attributes in their second attempt, likely as a result of learning from the initial experience. The dual-process framework used in this study highlighted how creative and conservative intentions influenced problem-solving approaches, with task familiarity tending to prompt conservative intentions, except among teens, young adults, and seniors, who showed greater divergent thinking in the second instance.

As an exploratory study, these findings offer preliminary insights rather than definitive conclusions, highlighting trends that merit further investigation. This research provides teachers, educational practitioners, and curriculum designers with a foundation for understanding how modular robotics can support CPS across diverse age groups. These findings are applicable to designing targeted educational strategies that encourage CPS development using modular robotics, including tools such as CreaCube and Lego robotics, in various educational contexts.

## Data Availability

The raw data supporting the conclusions of this article will be made available by the authors, without undue reservation.
